# Brief Maternal Separation Promotes Resilience to Anxiety-like and Depressive-like Behaviors in Female C57BL/6J Offspring with Imiquimod-Induced Psoriasis

**DOI:** 10.3390/brainsci12091250

**Published:** 2022-09-15

**Authors:** Lin Zhou, Zuotian Wu, Yixin Li, Ling Xiao, Huiling Wang, Gaohua Wang

**Affiliations:** 1 Department of Psychiatry, Renmin Hospital of Wuhan University, Jiefang Road No.238, Wuhan 430060, China; 2 Department of Neurology, Union Hospital, Tongji Medical College, Huazhong University of Science and Technology, Wuhan 430061, China

**Keywords:** depression, psoriasis, imiquimod, microglia, inflammation

## Abstract

Background: Psoriasis is a common chronic inflammatory skin disease that often causes depression. Early life experience affects brain development and relates to depression. Whether the effect of different MS protocols in early life on anxiety-like and depressive-like behaviors in female offspring with imiquimod (IMQ)-induced psoriasis is unknown. Methods: C57BL/6J mice were subjected to no separation (NMS), brief MS (15 min/day, MS15) or long MS (180 min/day, MS180) from postpartum days (PPD) 1 to PPD21. Then, 5% imiquimod cream was applied for 8 days in adults. Behavioral tests, skin lesions and hippocampal protein expression were also assessed. Results: We found significant psoriasis-like skin lesions in female mice following IMQ application, and mice showed anxiety-like and depressive-like behaviors. Further, increased microglial activation and decreased expression of neuroplasticity were detected in mice following IMQ application. However, after MS15 in early life, mice showed decreased anxiety-like and depressive-like behaviors, indicating resilience. Further, inhibited hippocampal neuroinflammation and increased neuroplasticity were detected. Conclusions: Collectively, this study confirms that brief MS confers resilience to the behavior deficits in female offspring with IMQ-induced psoriasis and reverses the activation of neuroinflammation and the damage of neuroplasticity injury.

## 1. Introduction

Psoriasis is a common chronic inflammatory skin disease. It often recurs and is difficult to cure. Patients with psoriasis are more prone to depression than patients with other skin diseases. The prevalence of depression in patients with psoriasis is estimated at 9% to 62% [1]. Depression can aggravate psoriasis’ recurrence and impair patients’ life quality, resulting in suicide and other serious consequences [2]. Therefore, the prevention of psoriasis-related depression is critical.

The hallmark of psoriasis is the appearance of acanthosis, erythema and inflammatory infiltrate. Psoriasis can be modeled in mice by daily topical application of Aldara cream containing 5% imiquimod (IMQ). IMQ is a classical TLR receptor agonist, which can activate immune cells through the TLR pathway, thus triggering a systemic immune response [3]. Recently, the IMQ-induced psoriasis model was found to cause depressive-like and anxiety-like behaviors in male mice [4].

Neuroinflammation is involved in the pathological mechanisms of depression [5]. The hippocampus is one of the brain regions that play an important pathological role in depression [6]. Microglia, as resident immune cells, are one of the most important inflammatory cells in the central nervous system (CNS). Active microglial cells in the hippocampus can promote activation of a series of regional inflammatory responses, including Nod-like receptor pyrin domain containing 3 (NLRP3), interleukin-1β (IL-1β) and interleukin-18 (IL-18). In the animal model of depression, emerging evidence indicates neuroinflammation can damage hippocampal neuroplasticity, including neuronal axon and dendritic tree retreat [7,8]. Collapsing response mediator protein 2 (CRMP2) has been recently considered a novel microtubule-associated protein, and it may have neuroprotective effects by modulating hippocampal neuroplasticity. The proteomic results in many rodent studies show CRMP2 is associated with stress-induced depression, with a significant decrease in hippocampal CRMP2 expression observed in rodents with depressive-like behaviors [9,10,11].

Early life experience has been proven to contribute to the development of depression [12,13]. Many studies in humans have indicated that exposure to separation, mistreatment and loss in early life increases the risk of psychiatric disorders such as depression, anxiety and drug abuse later in life [14,15,16]. Maternal separation (MS) is widely used in rodent animals to study the behavior changes and underlying neurobiological mechanisms in emotional disorders. In rodents, MS in early life has adverse effects on brain development, similar to its effects on humans. However, rodent research has shown that brief maternal separation in early life helps prevent the development of depression later in life [17,18]. Brief MS is believed to benefit the neural development of the offspring and enhance their stress resistance in adulthood [19,20]. Our previous studies have confirmed that offspring who experience maternal separation for 15 min daily in early life show resistance to chronic stress in adulthood; however, offspring who experience daily maternal separation for 180 min or more in early life show relative sensitivity to chronic stress in adulthood [17]. Another of our former studies also found that sex can affect behaviors in offspring [21]. Past research has mostly focused on the effects of MS on male offspring; few studies have focused on the effects on female offspring. It is important, then, to determine whether MS in early life affects behavior deficits in female offspring and, if so, its underlying mechanism.

Therefore, in this study, we firstly investigated the effect of IMQ on depressive-like and anxiety-like behaviors in female offspring. Then, we hypothesized that different MS protocols in early life would affect vulnerability or resilience to psoriasis-related behavioral phenotypes in female offspring. To test this, we developed a mouse model of different MS protocols and then applied IMQ to the animals to investigate the behavioral phenotypes. To further explore the neurobiological mechanism underlying this phenomenon, we then investigated microglial activation and some biomarkers of neuroinflammation and neuroplasticity in the hippocampus.

## 2. Methods and Animals

### 2.1. Animals

The adult female C57BL/6J mice (weighing 18–20 g) and pregnant C57BL/6J mice (weighing 26–30 g) used in the experiment were provided by the animal facility at Renmin Hospital of Wuhan University. All the animals were individually housed under standard conditions (room temperature 22 ± 1 °C, 55 ± 5% humidity, fed ad libitum) on a 12-h light/dark cycle (lights on from 6:00 a.m. to 6:00 p.m.) with free access to food and water. The experimental protocol was conducted in accordance with the Regulations of Experimental Animal Administration issued by the State Committee of Science and Technology of the People’s Republic of China and with the approval of the Ethics Committee in Renmin Hospital of Wuhan University.

### 2.2. Experimental Design

**Figure 1:**
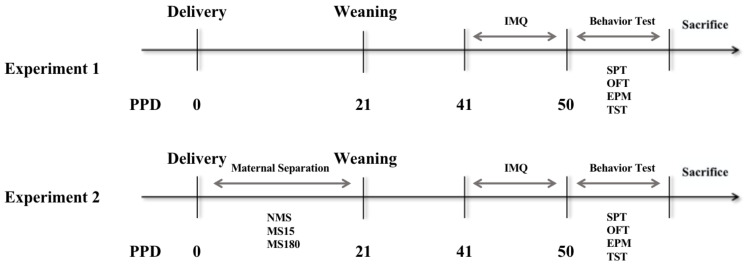
Experimental procedures. Experience 1: Female mice with no MS had imiquimod cream applied for 8 days consecutively. Experience 2: Female offspring were subjected to different PS protocols during lactation and then had imiquimod cream applied after adulthood. PPD, postpartum day; OFT, open field test; EPM, elevated plus maze; SPT, sucrose preference test; TST, tailed suspension test.

As shown in Figure 1, two experiments were conducted in this study. Experiment 1: The adult female mice who did not experience MS were randomly divided into two groups: the normal control (NC) group (*n* = 8) and the imiquimod model (IMQ) group (*n* = 8). Mice were depilated on the back skin 2 days before the treatment. Subsequently, 62.5 mg of 5% Imiquimod cream (Mingxin Pharmaceutical Co. Ltd., Chengdu, China) was applied daily on the backs of the IMQ mice group, while Vaseline (Lanlianfeitian Petrochemical Co., Ltd., Shijiazhuang, China) was applied on the mice of the NC group for 8 days consecutively. Experiment 2: Pregnant mice were randomly assigned to three groups: NMS group (no MS, NMS), MS15 group (15 min/day) and MS180 group (180 min/day). After delivery, offspring undertook different MS protocols during postpartum days (PPD) 1 through 21. After weaning, the litters were sexed, and the female offspring of each group were selected (*n* = 10). Then, the female mice were housed at 5 mice per cage under the same environmental conditions as previously described under “Animals”. After the offspring became adults at PPD 41, imiquimod cream was applied to all three groups. The behavioral tests were performed after applying imiquimod cream.

### 2.3. Maternal Separation (MS)

According to the previous research [17], the MS procedure was performed for 15 min/per day on the MS15 group (brief MS) and for 180 min/per day on the MS180 group (long MS) during PPD1 through 21. In contrast, the offspring in NMS group did not separate from their dams. When MS happened, the dams were first removed from the nest to a separate new cage, and the pups that remained in their original cage were placed in an incubator (maintained at a temperature of 30–32 °C). After separation, the dams were returned to the home cage. All other dams and pups were left undisturbed until weaning on postpartum day 21.

### 2.4. Sucrose Preference Test (SPT)

The SPT were performed after imiquimod cream treatment. SPT is a common method to assess depressive-like behavior in animals, especially anhedonia, the core symptom of depression. The SPT was carried out according to previous studies [22,23]. After 24 h of fasting and water deprivation, two bottles were provided to each mouse simultaneously, one bottle of tap water and the other of 1% sucrose solution. The two bottles of water were weighed. The fluid consumption was monitored for 1 h. After the monitoring, the bottles were removed and weighed. The sucrose preference ratio was calculated as the percentage of sucrose solution consumption out of the sum of tap water and sucrose solution consumption.

### 2.5. Open Field Test (OFT)

The OFT was performed after the SPT. The OFT is widely used to evaluate locomotor activity and depressive-/anxiety-like behavior in rodents. Decreased time in the center of the field means that the animal reveals anxiety-like and depressive-like behaviors [24]. The open field chamber was made of transparent plastic (50 cm × 50 cm). The chamber was divided into 9 square areas, and a 25 cm × 25 cm center square was defined as the center zone. Individual mice were gently placed in the central area of the cage, and the activities of the mice were recorded for 5 min with an overhead video-tracking system (Ethovision XT 11.5, Noldus, Amsterdam, The Netherlands). The floor and walls of the apparatus were thoroughly cleaned with 75% alcohol after each trial.

### 2.6. Elevated Plus Maze (EPM) Test

The EPM test was conducted after the OFT. The EPM test is widely used to detect anxiety-like behaviors. Animals always prefer closed arms; staying longer in closed arms means animals feel anxiety [25]. The EPM platform consisted of two open arms (35 cm × 5 cm) perpendicular to two closed arms (35 cm × 5 cm) of the same size with a small central square (5 cm × 5 cm) between the arms. The maze was raised 50 cm above the floor. Each animal was placed at the center of the maze, facing toward the open arms. The total number of entries into the open arms and the time spent in the open arms during the 5 min period were recorded using a Noldus video camera. The apparatus was cleaned with 75% ethanol after each trial.

### 2.7. Tail Suspension Test (TST)

The TST was performed after the EPM. The TST is usually used to detect depressive-like behaviors: when animals feel despair, they will stay immobile in the TST [26]. Mice were hung 40 cm above the floor by adhesive tape on the tip of the tail in a dark room. We recorded the total time that each mouse was immobile during 6 min. The tester was blinded to the group assignments of mice.

### 2.8. Psoriasis Area and Severity Index Score

A scoring system similar to the human Psoriasis Area and Severity Index ((PASI) was used to assess the disease severity of back skin daily. Scores were assigned according to the PASI scoring criteria: 0 = none; 1 = slight; 2 = moderate; 3 = severe; 4 = extremely severe. The scaling, erythema, skin thickness and cumulative score of each mouse were recorded and analyzed. A cumulative score was generated from the three mentioned parameters. The researcher was blinded to the group assignments of mice.

### 2.9. Tissue Collection

After the behavioral tests, half of the mice in the same group were randomly anesthetized with 1% pentobarbital (35 mg/kg). After decapitation, the brains were immediately removed and rapidly frozen on dry ice and stored at −80 °C; further, the hippocampus was removed with microscissors for Western blot analyses. The other half were deeply anesthetized with 1% pentobarbital. After heart perfusion with saline, followed by 40 mL 4% paraformaldehyde, brains were quickly removed and post-fixed in 4% paraformaldehyde for 4–6 h and subsequently cryoprotected separately in 35% sucrose. The skin lesions on the backs of mice were also carefully removed for the subsequent hematoxylin and eosin (H&E) and immunohistochemical staining.

### 2.10. Western Blot Analysis

The whole hippocampus of each mouse was mixed to detect Western blot. The total amounts of proteins were detected by the bicinchoninic acid (BCA) method (Beyotime, Shanghai, China). Proteins (20 μg) were separated by SDS-PAGE on 10% polyacrylamide gels and transferred electrophoretically to polyvinylidene fluoride (PVDF) membranes with the TGX Stain-FreeTM FastCastTM Acrylamide Kit (Bio-Rad). The membranes were blocked with 5% nonfat dry milk in TBS + 0.1% Tween-20 (TBST) for 1 h and incubated overnight at 4 °C with the following primary antibodies in Bond Primary Antibody Diluent: rabbit anti-NLRP3 (dilution 1:1000, ab263899, Abcam, Cambridge, UK), rabbit anti-caspase-1 (dilution 1:1000, ab179515, Abcam, Cambridge, UK), rabbit anti-IL-18 (dilution 1:1000, ab191860, Abcam, Cambridge, UK), rabbit anti-IL-1β (dilution 1:1000, ab234437, Abcam, Cambridge, UK), rabbit anti-CRMP2 (dilution 1:20,000, ab129082, Abcam, Cambridge, UK), mouse anti-α-tubulin (dilution 1:5000, ab7291, Abcam, Cambridge, UK) and rabbit anti-GAPDH (dilution 1:1000, ab181602, Abcam, Cambridge, UK). After being washed thrice in TBST, the membranes were incubated with secondary antibodies (HRP-labelled goat anti-rabbit IgG, 1:5000; goat anti-mouse IgG, 1:10,000, Abcam, Cambridge, UK) at room temperature for 1 h. The proteins were detected by the Bio-Rad ChemiDoc Touch Image System (Bio-Rad), and the results were standardized to the GAPDH band at 37 kDa as an internal control with Bio-Rad software.

### 2.11. Immunofluorescence

For immunofluorescence staining, samples were dehydrated by graded ethanol, embedded in paraffin and sliced into 5 μm thick sections. Iba1 is a marker of microglial activation, and the increased expression of IBA1 denotes increased microglial activation. Sections were incubated at 4 °C overnight with Iba1 antibody (Abcam, ab178847, Cambridge, UK). After being rinsed in PBS thrice (5 min/time), the sections had secondary antibodies goat-anti-rabbit IgG (Cy3) (Abcam; 1:200) applied to them at room temperature for 1 h. Then, they were rinsed thrice (5 min/time) in 100 mM PBS. After mounting with Antifade Mounting Medium with DAPI (VECTOR, H-1200), pictures were obtained with the automatic fluorescence microscope (Olymplus, Tokyo, Japan). The IOD of Iba1 was measured by Image J, version 8.0 (National Institutes of Health, Bethesda, MD, USA).

### 2.12. Histopathological Examination and Immunohistochemical Staining

For histological staining, 5 μm thick tissue sections were cut from the skin paraffin sections and stained with H&E to observe pathological changes in the mice’s skin lesions. The histopathological changes were observed and photographed under a microscope (Olympus, Tokyo, Japan). The epidermal thickness was measured by Image Pro Plus software, version 8.0 (Media Cybernetics, Rockville, MD, USA). For immunohistochemical staining, the tissue sections (5 μm) were stained with rabbit anti-NLRP3 (dilution 1:200, ab263899, Abcam, Cambridge, UK). The expression at the positive spots was evaluated by microscopy (Olympus,Tokyo, Japan). The positive expression was measured by Image J software, version 8.0 (Rawak Software Inc, Stuttgart, Germany).

### 2.13. Statistical Analysis

The results are presented as the means ± standard errors of the means (SEM). SPSS software, version 22 (IBM Corp., Armonk, NY, USA) and GraphPad Prism software version 8.0 for Windows (GraphPad Software, Inc., San Diego, CA, USA) were used for the analysis. The Kolmogorov–Smirnov normality test and the variance homogeneity test were used to ensure that the normality and variance heterogeneity assumptions were met. Statistical comparisons were made using a *t*-test or one-way analysis of variance (ANOVA) followed by Tukey’s post hoc test. Statistical significance was considered if the *p*-value was < 0.05.

## 3. Results

### 3.1. The Skin Lesions of Psoriasis-like Female Mice

**Figure 2:**
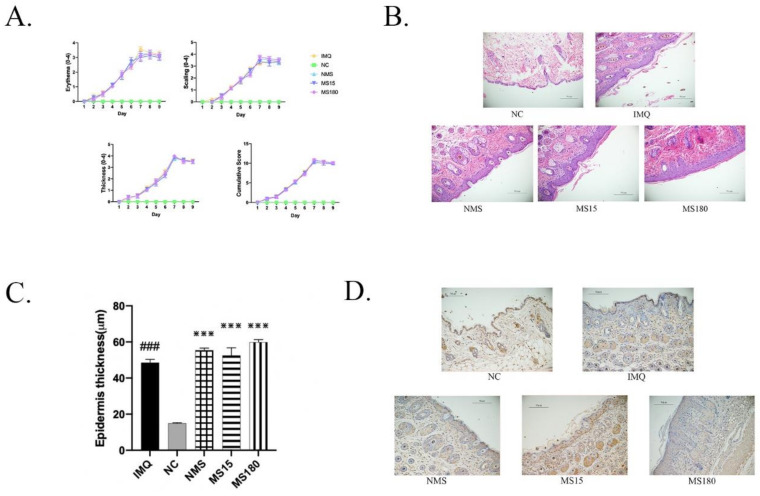
(**A**) The trend of PASI score in mice with skin lesions, which includes erythema score, scales score, thickness score, and cumulative score. Cumulative score = (Erythema score + Scales score + Thickness score). (**B**) Histopathological alterations in skin lesions following H&E staining (200), scale bar = 50 m. (**C**) Mice epidermal thickness in each group. ### *p* < 0.001 vs. the NC; *** *p* < 0.001 vs. the NMS; *n* = 10. (**D**) The immunohistochemical changes of NLRP3 in skin lesions (×200), scale bar = 50 µm.

Firstly, we detected the changes in hypertrophic scales, erythema, thickness and inflammation infiltration in the skin lesions of female mice induced by imiquimod after 9 days. As shown in Figure 2, the PASI scores of the IMQ group were significantly increased compared to the NC group (*p* < 0.0001) on day 9, indicating that the psoriasis-like skin lesion model was successfully induced. Then, we carried out pathological H&E staining, finding that epidermal thickness in the IMQ group was significantly increased (*p* < 0.0001) compared to the NC group. We also carried out immunohistochemical testing, with NLRP3 being found to be increasingly expressed in the back skin lesions of the IMQ group compared to the NC group (*p* < 0.0001), but there existed no differences among the NMS, MS15 and MS180 groups.

### 3.2. Anxiety and Depressive-like Behaviors in Psoriasis-like Female Mice

**Figure 3:**
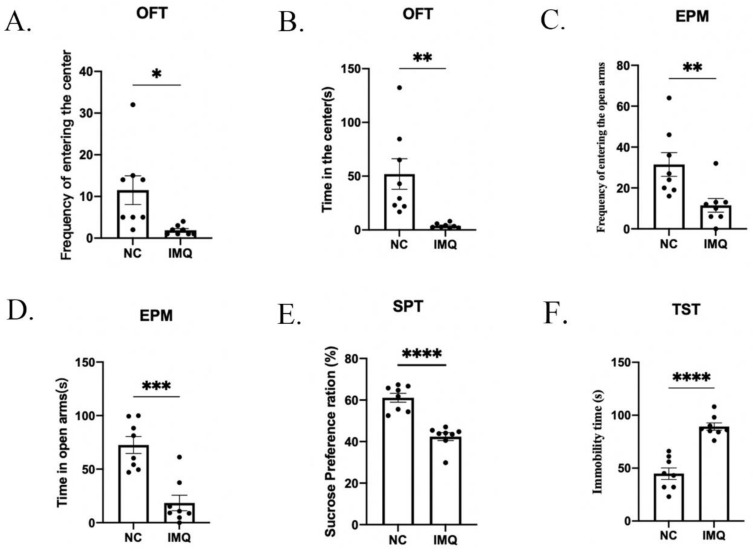
Behavioral deficits in IMQ induced psoriasis-like female mice without experiencing MS. (**A**) The frequencies of entering the center; (**B**) time in the center in the OFT. (**C**) The frequencies of entering the center; (**D**) time in the open arms in the EPM. (**E**) The sucrose preference ratio in the SPT. (**F**) Immobility time in the TST. Each value represents the mean ± SEM. *n* = 8 animals per group. Two groups (NC, IMQ) were analyzed using *t*-test (* *p* < 0.05, ** *p* < 0.01, *** *p* < 0.001 and **** *p* < 0.0001).

After IMQ modeling, we investigated the depressive- and anxiety-like behaviors in female mice using the OFT, EPM, SPT and TST. As shown in Figure 3A,B, the frequency of entering the center and time spent in the center were significantly decreased in the IMQ group compared to the NC group (the frequency of entering the center: t = 2.791, *p* < 0.0001; time spent in the center: t = 3.279, *p* < 0.0001). As shown in Figure 3C,D, compared with the NC group, the IMQ group showed significant decreases in the frequency of entering the open arms and time spent in the open arms (the frequency of entering the open arms: t = 2.747, *p* < 0.05; time spent in the open arms: t = 4.027, *p* < 0.001). As shown in Figure 3E,F, mice in the IMQ group showed a lower sucrose preference ratio (t = 6.533, *p* < 0.0001) and more immobility time in the TST compared with the NC group (t = 8.016, *p* < 0.0001).

### 3.3. Neuroinflammation Changes in Psoriasis-like Female Mice

**Figure 4:**
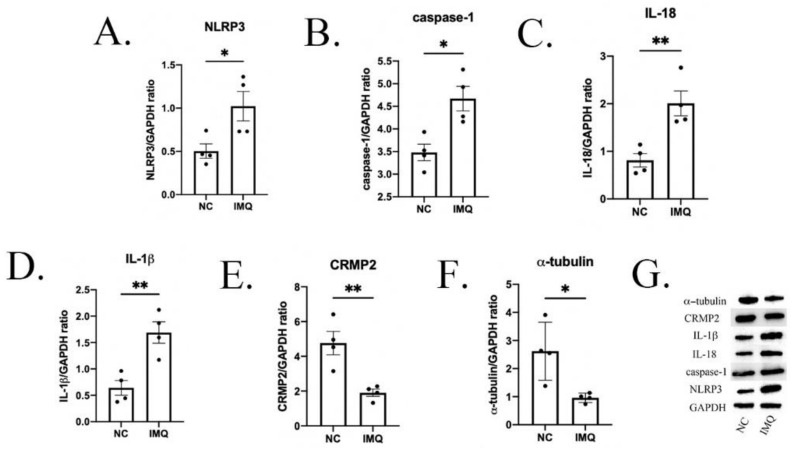
Hippocampal protein levels of neuroinflammation and neuroplasticity biomarkers in mice. Western blot analysis of (**A**) NLRP3, (**B**) caspase-1, (**C**) IL-18, (**D**) IL-1β, (**E**) CRMP2, and (**F**) α-tubulin protein expression. (**G**) Densitometry analyses of the bands. Each value represents the mean ± SEM. *n* = 4 animals per group. Two groups (NC, IMQ) were analyzed using *t*-test (* *p* < 0.05, ** *p* < 0.01).

We investigated several neuroinflammation biomarkers in the hippocampus to explore the possible mechanisms of behavior deficits in psoriasis-like mice. As shown in Figure 4A–D, compared to the NC group, the protein expression of NLRP3, caspase-1, IL-18 and IL-1β in the IMQ group were significantly higher (NLRP3: t = 2.745, *p* < 0.05; caspase-1: t = 3.614, *p* < 0.05; IL-18: t = 4.263, *p* < 0.01; IL-1β: t = 4.008, *p* < 0.01).

**Figure 5:**
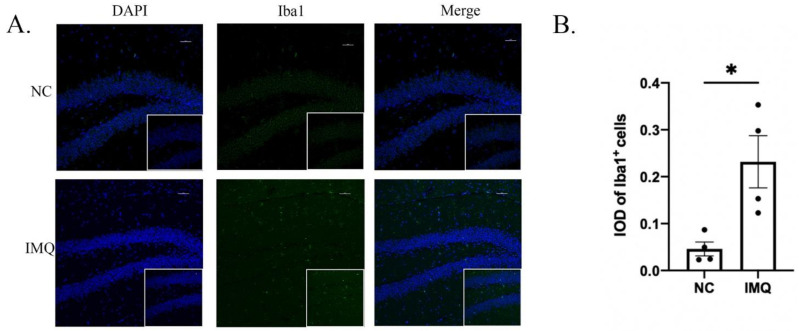
Iba1 immunoreactivity of microglia in the hippocampus of female mice. (**A**) Representative images of labeling for Iba1. (**B**) The integral optical density (IOD) of Iba1^+^ cells in hippocampus (×200), scale bar = 50 µm. Each value represents the mean ± SEM. *n* = 4 animals per group. Two groups (NC, IMQ) were analyzed using *t*-test (* *p* < 0.05).

As shown in Figure 5A,B, the immunofluorescence results for iba1 demonstrated that the activation of iba1 in the IMQ group was significantly higher than that in the NC group (t = 3.221, *p* < 0.05).

### 3.4. Neuroplasticity Changes in Psoriasis-like Female Mice

Then, we detected the expression of several typical biomarkers of neuroplasticity. As shown in Figure 4E,F, compared with the NC group, the protein expression of CRMP2 and α-tubulin was significantly lower in the IMQ group (CRMP2: t = 4.054, *p* < 0.01; α-tubulin: t = 3.172, *p* < 0.05).

### 3.5. Brief MS Promoted Resilience to Behavior Deficits in Psoriasis-like Female Offspring

**Figure 6:**
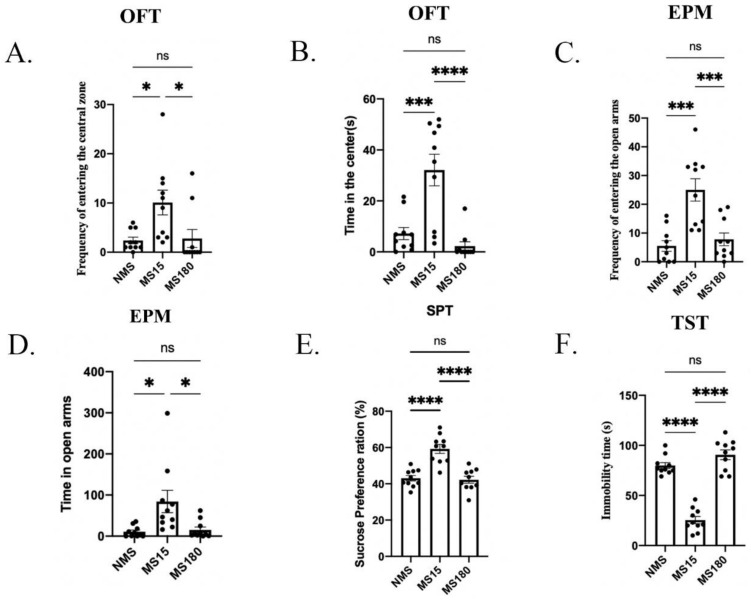
Brief PS during lactation conferred stress resilience to behavioral deficits in IMQ-induced psoriasis-like female mice. (**A**) The frequencies of entering the center; (**B**) time in the center in the OFT. (**C**) The frequencies of entering the center; (**D**) time in open arms in the EPM. (**E**) The sucrose preference ratio in the SPT. (**F**) Immobility time in the TST. Each value represents the mean ± SEM. *n* = 10 animals per group. Three groups were analyzed using ANOVA followed by a post hoc analysis (ns *p* > 0.05, * *p* < 0.05, *** *p* < 0.001 and **** *p* < 0.0001).

In the second experiment, we explored the effects of different types of maternal separation on female offspring with a psoriasis-like disease. In the OFT, significant differences were found among the three groups in the frequency of entering the center (F (2, 27) = 5.582, *p* = 0.0094, Figure 6A) and the time spent in the center (F (2, 27) = 16.43, *p* < 0.0001, Figure 6B). In the following post hoc analysis, the frequency of entering the center was found to be significantly higher in the MS15 group than that in the NMS group and MS180 group (Both *p* < 0.05), but there was no difference between the NMS group and MS180 group (*p* = 0.9870). The time spent in the center for the MS15 group was greater than that for the NMS group and MS180 group (MS15 + CRS and NMS + CRS, *p* < 0.001, MS15 + CRS and MS180 group, *p* < 0.0001), but no significant difference was observed between the MS180 and NMS groups (*p* = 0.6532).

In the EPM test, significant differences were found among the three groups in the frequency of entering the open arms (F (2, 27) = 14.52, *p* < 0.0001, Figure 6C) and the time spent in the open arms (F (2, 27) = 6.325, *p* = 0.0056, Figure 6D). In the following post hoc analysis, the frequency of entering the open arms was found to be significantly higher in the MS15 group than in the NMS group and MS180 group (MS15 + CRS and NMS + CRS, *p* = 0.0001, MS15 + CRS and MS180 group, *p* < 0.0001), but no significant difference was observed between the MS180 and NMS group (*p* = 0.8311). The time spent in the open arms for the MS15 group was significantly higher than that for the NMS group and MS180 group (MS15 + CRS and NMS + CRS, *p* < 0.05, MS15 + CRS and MS180 group, *p* < 0.05), but no significant difference was observed between the MS180 and NMS group (*p* = 0.9784).

In the SPT, significant differences were found among the three groups in the frequency of entering the open arms (F (2, 27) = 23.08, *p* < 0.0001, Figure 6E). In the following post hoc analysis, the sucrose preference ratio of the MS15 group was found to be lower compared to that of the NMS group and MS180 group (MS15 + CRS and NMS + CRS, *p* < 0.0001, MS15 + CRS and MS180 group, *p* < 0.0001), while no difference was found between the NMS group and MS180 group (*p* = 0.9543).

In the TST, significant differences were found among the three groups in the immobility time (F (2, 27) = 80.89, *p* < 0.0001, Figure 6F). In the following post hoc analysis, the immobility time of the MS15 group was lower compared to the NMS group and MS180 group (MS15 + CRS and NMS + CRS, *p* < 0.0001, MS15 + CRS and MS180 group, *p* < 0.0001), while no difference was found between the NMS group and MS180 group (*p* = 0.1411).

### 3.6. MS15 Protocol Inhibited Activation of Neuroinflammation in the Hippocampus of Female Mice Resilience to Behavior Deficits

**Figure 7:**
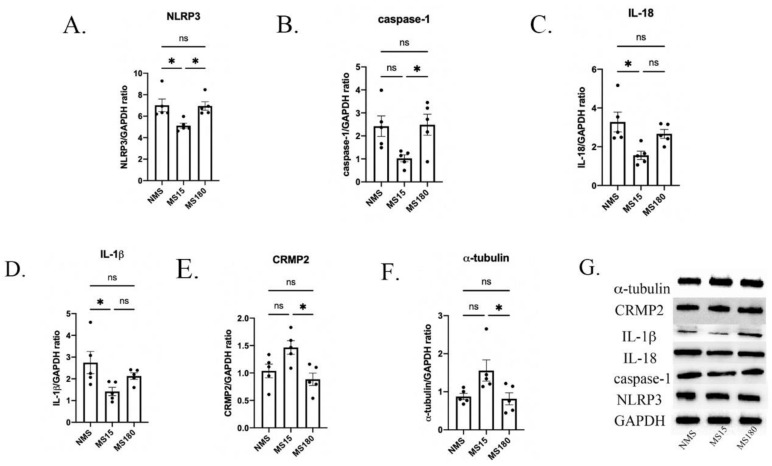
Hippocampal protein levels of neuroinflammation and neuroplasticity biomarkers in IMQ-induced psoriasis-like female mice with different MS protocols. Western blot analysis of (**A**) NLRP3, (**B**) caspase-1, (**C**) IL-18, (**D**) IL-1β, (**E**) CRMP2, and (**F**) α-tubulin protein expression. (**G**) Densitometry analyses of the bands. NMS, MS15 and MS180 groups were concluded. Each value represents the mean ± SEM. *n* = 5 animals per group. All 3 groups were analyzed using ANOVA followed by a post hoc analysis (ns *p* > 0.05, * *p* < 0.05).

We then explored whether different types of maternal separation affected the activation of several inflammatory cytokines in the hippocampus of psoriasis-like female offspring. As shown in Figure 7A–D, significant differences were found in the protein expression of NLRP3, caspase-1, IL-18 and IL-1β among the three groups (NLRP3: F (2, 12) = 6.306, *p* = 0.0134; caspase-1: F (2, 12) = 4.777, *p* = 0.0298; IL-18: F (2, 12) = 5.136, *p* = 0.0245; IL-1β: F (2, 12) = 4.127, *p* = 0.0433).

Then, in post hoc analysis, the protein expression of NLRP3 in the MS15 group was found to be lower than that in the NMS group and MS180 group (Both *p* < 0.05), but no difference was found between the NMS group and MS180 group (*p* = 0.9943). The protein expression of caspase-1 in the MS15 group was lower than in the MS180 group (*p* < 0.05), but no difference was found between the NMS group and MS15 group (*p* = 0.0547), and no difference was found between the NMS group and MS180 group either (*p* = 0.9925). Further, compared with the NMS group, the protein expression of IL-18 and IL-1β was significantly lower in the MS15 group (Both *p* < 0.05), but no difference was found between the MS15 group and MS180 group, and no differences were found between the NMS group and MS180 group either.

**Figure 8:**
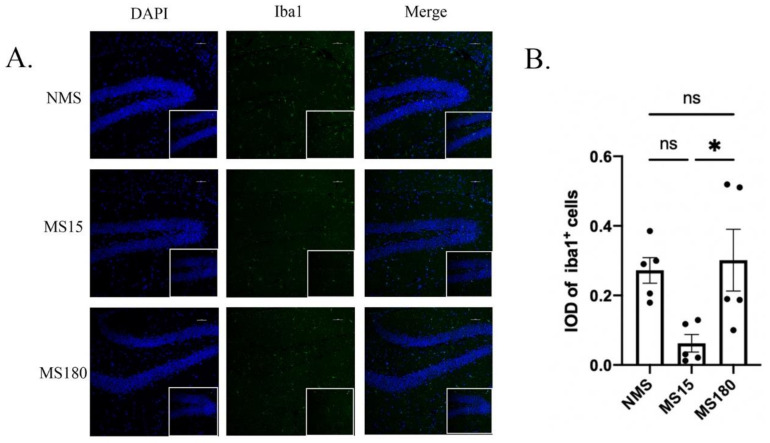
Iba1 immunoreactivity of microglia in hippocampus of IMQ-induced psoriasis-like female mice with different MS protocols. (**A**) Representative images of labeling for Iba1. (**B**) The integral optical density (IOD) of Iba1^+^ cells in hippocampus (×200), scale bar = 50 µm. NMS, MS15 and MS180 groups were concluded. Each value represents the mean ± SEM. *n* = 5 animals per group. All 3 groups were analyzed using ANOVA followed by a post hoc analysis (ns *p* > 0.05, * *p* < 0.05).

As shown in Figure 8A–B, significant differences were found in the IOD of Iba1 + cells among the three groups (F (2, 12) = 5.175, *p* = 0.0240). Then, in the post hoc analysis, the IOD of iba1 + cells was found to be lower in the MS15 group compared to the MS180 group (*p* < 0.05), but there was no difference between the NMS group and MS15 group, nor between the NMS group and MS180 group.

### 3.7. MS15 Protocol Inhibited Hippocampal Neuroplastic Injury in Female Mice Resilience to Behavior Deficits

We further explored the effect of different types of maternal separation on the hippocampal neuroplasticity of psoriasis-like female offspring. As shown in Figure 7E–F, significant differences were found in the protein expression of CRMP2 and α-tubulin among the three groups (CRMP2: F (2, 12) = 6.257, *p* = 0.0138; α-tubulin: F (2, 12) = 4.668, *p* = 0.0317).

Then, in post hoc analysis, the protein expression levels of CRMP2 and α-tubulin were both higher in the MS15 group compared to the MS180 group (both *p* < 0.05), but there was no difference between the NMS group and MS15 group, nor between the NMS group and MS180 group.

## 4. Discussion

This study firstly explored the effect of IMQ on behavioral phenotypes in female adult mice who did not experience maternal separation in early life. Then, it investigated the effect of different types of maternal separation on depressive- and anxiety-like behaviors in female offspring with IMQ-induced psoriasis, as well as the underlying mechanism with regard to neuroinflammation and neuroplasticity. We firstly found that the IMQ-induced psoriasis-related female mice showed depressive- and anxiety-like behaviors, which were associated with microglial activation, the up-regulation of NLRP3, Caspase-1, IL-18, and IL-1β, and the down-regulation of CRMP2 and α-tubulin. Then, we found that the MS15 procedure promoted resilience to behavioral deficits in female mice with psoriasis: microglial activation and the NLRP3 pathway were inhibited, and CRMP2 and α-tubulin were increased in the MS15 mice.

We firstly explored behavioral responsiveness in mice using the OFT, EPM, SPT and TST. In this study, after IMQ application, the mice showed significant decreases in the time spent in the center/open arms and the frequency of entering the center/open arms in the OFT and EPM, decreased sucrose preference in the SPT and increased immobility in the TST with psoriasis-like skin lesions on their backs. These indicated that the female mice with psoriasis-like skin lesions showed significant anxiety-like and depressive-like behaviors, which was consistent with a recent study’s findings [4].

Inflammation has been proven to play an important role in the pathology of depression. NLRP3 is an intracellular multi-protein complex that links the perception of danger to the proteolytic activation of pro-inflammatory cytokines. When NLRP3 is activated, it activates Caspase-1 and promotes the conversion of pro-IL-1β into bioactive IL-1β, which causes an inflammatory response. IL-18 is a member of the cytokine interleukin family, which is widely expressed in activated microglia, astrocytes and neuronal cells. Microglia are key immune cells in the brain. In response to stress, microglia change into the activated phenotype, which stimulates the activation of NLRP3 and the secretion of downstream inflammatory factors, including IL-18 and IL-1β [27]. In our study, we firstly detected the expression of neuroinflammation in the hippocampus of mice without maternal separation and found significantly increased expression levels of NLRP3, Caspase-1, IL-1β, and IL-18, and the upregulation of microglia activation, indicating hippocampal neuroinflammation was increased in mice with psoriasis-like skin lesions. Previous studies have also revealed that the activation of NLRP3 plays an important role in different animal models with anxiety-like and depressive-like behaviors [28,29]. Furthermore, the inhibitors of NLRP3 have often been exhibited to prevent depressive-like behaviors in animals [30,31]. Much research has reported significant activation of microglia in depressive animal models induced by LPS injection [30,32]; further, in stress-induced non-inflammatory depression models, the activation of microglia has also been found to play an important role [33], which is consistent with our findings.

The activation of neuroinflammation and the release of some inflammatory factors promote damage to neuroplasticity. α-tubulin is one of the main components of the microtube system, and CRMP2 interacts directly with α-tubulin to modulate its function [34,35]. CRMP2 can affect neuroplasticity through its interaction with α-tubulin [36]. CRMP2 is also related to the mechanism of the repair and regeneration of adult brain neurons [37]. Many previous studies found that in mice with depressive-like behaviors induced by chronic stress, the expression of CRMP2 was significantly decreased, and the neuroplasticity in the hippocampus showed significant injury [10,38]; however, the neuroplasticity in the hippocampus was enhanced after some measures that can improve or prevent depressive-like behaviors were taken [39,40,41]. In this study, we found that there was a significant down-regulation of CRMP2 and α-tubulin in the hippocampus of psoriasis-like mice without maternal separation, which represented damage to the neuroplasticity in the hippocampus.

Maternal separation is a widely used paradigm to study the effect of early-life experience on brain development and resilience to psychopathology in rodents. In this study, after we found behavioral deficits in psoriasis female mice, we further explored the effects of different MS procedures on the behavior responsiveness in psoriasis mice. Notably, the psoriasis mice under the MS15 procedure showed resilience to behavioral deficits. Specifically, compared with NMS, MS15 led to increased time in the center and an increased frequency of entering the center in the OFT and EPM, increased sucrose preference in the SPT and decreased immobility time in the TST, which indicated the phenomenon of resilience. However, the psoriasis mice under the MS180 procedure showed significant behavioral deficits compared with those without maternal separation. In recent years, the effect of resilience on the prevention of depression has attracted increasing attention [42]. Our previous study showed that mice with brief MS revealed resistance to the emergence of anxiety-like and depressive-like behaviors caused by chronic stress re-exposure later in life, which is consistent with the present results [17]. In the present study, MS180 did not aggravate depressive-like behaviors in mice following IMQ, which means that maternal separation only increased the sensitivity of mice to stress but not the severity of depressive-like behaviors.

Interestingly, the relationship between stress exposure and the stress response is subtle. Moderate stress exposure can promote positive coping responses and produce stress resistance. In this study, when pups experienced prolonged daily maternal deprivation after birth, they showed higher sensitivity to subsequent stressors in adulthood. However, if stress exposure in early life is less severe, it can have the effect of promoting recovery, a process known as stress inoculation. Studies have shown that pups exposed to moderate life stress after birth displayed lower plasma levels of corticotropin-releasing hormone and an attenuated stress-induced increase in plasma corticosterone than undisturbed pups and pups exposed to prolonged maternal separation [43]. Additionally, the expression of plasma glucocorticoids has been proven to facilitate NLRP3 induction and inflammasome formation and thus promote activation of the inflammatory response and the release of pro-inflammatory cytokines such as IL-1β [44].

Notably, in our study, there were no significant differences in the activation of NLRP3 in skin lesions in mice which underwent different MS procedures. However, after brief MS in early life, mice with psoriasis-like skin lesions showed decreased activation of microglia and decreased expression of the NLRP3/Caspase-1/IL-1β pathway in the hippocampus, which suggests that even though brief MS did not inhibit the activation of skin inflammation, brief MS may have prevented depressive-like behaviors by inhibiting microglial activation in adulthood. Our previous study found that male mice with long maternal separation and mice which did not experience maternal separation all had increased sensitivity to chronic stress and inflammatory activation in adulthood, while mice with brief maternal separation showed resilience to chronic stress, results which are consistent with this study. Other studies have shown that brief maternal separation can damage the development and maturation of microglial cells, which may be the potential mechanism that the maternal separation procedure affects, leading to the activation of neuroinflammation in adulthood [45,46]. Meanwhile, corticosterone levels and oxidative stress activation have been found to be significantly higher in rodents after long maternal separation [47]. In another study, neuronal development and maturation in the hippocampus were impaired in female offspring with long maternal separation, leading to deficits in learning and memory, whereas neuronal development was normal in offspring with brief maternal separation [48]. A clinical study also showed that children who were separated from their mothers for a long period of time had an increased burden of inflammation and a higher risk of psychiatric disease [49].

Enhanced neuroplasticity has been proven to prevent and improve depressive-like behaviors in rodents. The activation of inflammation has been found to accelerate damage to neuroplasticity [50]. However, anti-depressive drugs have been proven to improve neuroplasticity, reversing depressive-like behaviors [51]. Our previous study found that neuroplasticity was involved in resilience in LPS-induced acute depressive-like behaviors in dams [52]. Further, early life experience has been found to play an important role in the development of neuroplasticity [53,54]. Long maternal separation was found to accelerate damage to hippocampal neuroplasticity in mice, promoting the development of depression, while brief maternal separation improved neuroplasticity in mice, promoting resistance to depressive-like and anxiety-like behaviors induced by chronic stress [17]. Then, in this study, we found that brief maternal separation inhibited neuroplasticity injury in female adult mice following IMQ application, and brief maternal separation inhibited microglial activation. We propose that brief maternal separation may restore neuroplasticity by inhibiting the activation of microglia. This provides a possible neurobiological mechanism for resilience to depression.

The realization of brain function depends on changes in neural activity, which is based on synaptic plasticity. Microglia have been found to regulate synaptic plasticity. Recent studies have confirmed the interaction between neurons and microglia, which can promote synapse remodeling in the hippocampus [55]. Meanwhile, the activation of microglia can promote damage to α-tubulin, leading to the occurrence of mental diseases [56]. Activated microglia have been found to cause tau-induced synaptic loss and memory deficits [57]. NLRP3 has been found to cause deficits in synaptic plasticity in animal models of Alzheimer’s disease [58]. Peripheral monocytes have been found to cause changes in cortical neurons and synapses through NLRP3-dependent IL-1β, and reducing the peripheral active NLRP3 inflammasome can reverse the defects in neurons and synapses in the brain [59]. The protective effect of CRMP2 on neuroplasticity may contribute to resistance to neuroinflammation [60]. However, more research is needed to investigate the underlying mechanism.

## 5. Conclusions

In our study, mice with brief MS in early life showed resilience to psoriasis-related anxiety and depressive-like behaviors in female mice, with neuroinflammation activation and neuroplasticity injury in the hippocampus inhibited. In both humans and mammals, maternal separation may affect the development of neuroplasticity, neuroinflammation and other mechanisms and change the process of neural development, thus changing the body’s resistance to stress in adulthood. Our study provides further evidence for the beneficial effect of brief MS and the potential mechanism underlying this, which may be a new strategy for the prevention of depression.

## Data Availability

Data will be made available on request.
